# Functional Divergence of the Closely Related Genes PhARF5 and PhARF19a in *Petunia hybrida* Flower Formation and Hormone Signaling

**DOI:** 10.3390/ijms252212249

**Published:** 2024-11-14

**Authors:** Yiqing Ding, Yunfeng Miao, Lingxuan Huang, Huijun Zhu, Wenle Li, Wei Zou, Shumin Yu, Bin Dong, Shiwei Zhong

**Affiliations:** 1 School of Landscape Architecture, Zhejiang Agriculture and Forestry University, Hangzhou 311300, China; dyq202411@163.com (Y.D.); 2019105052024@stu.zafu.edu.cn (Y.M.); hlx851390605@gmail.com (L.H.); zhuhuijun@stu.zafu.edu.cn (H.Z.); liwenle1234@126.com (W.L.); zzww5125@126.com (W.Z.); yushymin2023@163.com (S.Y.); 2 Zhejiang Provincial Key Laboratory of Germplasm Innovation and Utilization for Garden Plants, Hangzhou 311300, China; 3 Key Laboratory of National Forestry and Grassland Administration on Germplasm Innovation and Utilization for Southern Garden Plants, Hangzhou 311300, China

**Keywords:** *Petunia hybrida*, flower development, auxin signaling, *ARFs*, phytohormone

## Abstract

The ARF gene family plays a vital role in regulating multiple aspects of plant growth and development. However, detailed research on the role of the ARF family in regulating flower development in petunia and other plants remains limited. This study investigates the distinct roles of *PhARF5* and *PhARF19a* in *Petunia hybrida* flower development. Phylogenetic analysis identified 29 PhARFs, which were grouped into four clades. VIGS-mediated silencing of *PhARF5* and *PhARF19a* led to notable phenotypic changes, highlighting their non-redundant functions. *PhARF5* silencing resulted in reduced petal number and limb abnormalities, while *PhARF19a* silencing disrupted corolla tube formation and orientation. Both genes showed high expression in the roots, leaves, and corollas, with nuclear localization. The transcriptomic analysis revealed significant overlaps in DEGs between *PhARF5* and *PhARF19a* silencing, indicating shared pathways in hormone metabolism, signal transduction, and stress responses. Phytohormone analysis confirmed their broad impact on phytohormone biosynthesis, suggesting involvement in complex feedback mechanisms. Silencing *PhARF5* and *PhARF19a* led to differential transcription of numerous genes related to hormone signaling pathways beyond auxin signaling, indicating their direct or indirect crosstalk with other phytohormones. However, significant differences in the regulation of these signaling pathways were observed between *PhARF5* and *PhARF19a*. These findings reveal the roles of *ARF* genes in regulating petunia flower development, as well as the phylogenetic distribution of the PhARFs involved in this process. This study provides a valuable reference for molecular breeding aimed at improving floral traits in the petunia genus and related species.

## 1. Introduction

Auxin, one of the most important phytohormones, elicits responses with a great ability to modulate diverse aspects of plant growth and development depending on the cellular and developmental context through the auxin signaling pathway. While cytoplasmic auxin networks are present in the endoplasmic reticulum and plasma membranes, the classical nuclear auxin signaling pathway remains the most thoroughly characterized [1,2]. This pathway operates through a mechanism whereby auxin-dependent degradation of auxin/indoleacetic acid (Aux/IAA) transcriptional repressors results in gene activation, contingent on the cellular spatio-temporal context. This degradation process relies on the F-box protein TIR1, which binds to Aux/IAA in the presence of auxin, thereby conferring target specificity that ultimately results in the ubiquitination and subsequent degradation of Aux/IAA [3,4,5]. Aux/IAA repressors form heterodimers with auxin response factors (ARFs), inhibiting their transcriptional activation. Consequently, auxin-stimulated proteolysis of Aux/IAA repressors results in the release and activation of ARFs, thereby initiating the expression of early auxin-responsive genes [6]. The expression of hundreds of genes is altered within minutes, driven by concentration gradients. Among these, early auxin-inducible genes fall into three primary families: Small Auxin-up RNA (SAUR), Gretchen Hagen 3 (GH3), and Aux/IAA [7,8,9].

ARFs are crucial for regulating early auxin-responsive gene transcription within the auxin signaling pathway by binding specifically to auxin response elements to direct target gene expression [10]. Each ARF contains a conserved N-terminal DNA-binding domain (DBD), a plant-specific B-type, which recognizes auxin response elements (AuxRE) and requires additional N-terminal and C-terminal amino acids [11]. The C-terminal domain (CTD), necessary for dimerization, resembles the III and IV domains of Aux/IAA, enabling protein–protein interactions through heterodimerization with Aux/IAA or homodimerization with other ARFs, although this domain is incomplete in many ARFs [11,12]. The middle region (MR), situated between the DBD and CTD, facilitates transcriptional activation through enrichment in amino acids like glutamine (Q), leucine (L), and serine (S) or repression through enrichment in proline (P), serine (S), glycine (G), and leucine (L) [13].

*ARFs* exhibit multiple biological functions, including the regulation of growth and development in roots, leaves, flowers, fruit ripening, senescence, and responses to biotic and abiotic stimuli [14]. In *Arabidopsis*, *AtARF19*, which functions redundantly with *AtARF7*, is crucial for regulating lateral root formation, as its mutants exhibit slightly reduced lateral root numbers. *AtARF5* also likely regulates lateral root formation redundantly through the transcriptional activation of *AtARF7/19* downstream target genes [15,16,17,18]. Additionally, *ARF19* affects leaf cell expansion in *Arabidopsis* and regulates phytohormones to influence leaf size in *Oryza sativa* [18,19,20]. And its overexpression lines also exhibit a decreased height phenotype, reducing the yield of *O. sativa* [21]. *ARF19* also responds to drought and cold stress in *O. sativa* and to alkalinity at the transcriptional level in *Solanum lycopersicum* [22,23,24]. Like *ARF19*, *AtARF5* targets the promoters of *Wusche-related Homeobox 1* (*WOX1*) and *Pressed Flower* (*PRS*) antagonistically, affecting leaf flattening and participating in viral defense in *O. sativa* [25,26]. In contrast, *ARF5* regulates fruit setting and development by modulating auxin and GR signaling in *S. lycopersicum*, and as the *ARF MONOPTEROS* in *Arabidopsis*, it is crucial for flower primordium initiation [27,28,29]. Therefore, in *Arabidopsis*, homologous genes such as *ARF5*, *ARF7*, and *ARF19* are often found to have similar, and thus redundant, biological functions, with research on their role in flower development regulation being relatively limited. Moreover, although these *ARFs* share similar functions, they exert diverse effects on downstream genes, and the similarities and differences in their transcriptional and metabolic impacts warrant further exploration. Other *ARFs* regulating flower development in *Arabidopsis* include *AtARF2* and *AtARF3*, whose single mutants cause abnormal flower morphology, such as defects in floral organ number and pistil patterns. *AtARF2* is also a positive regulator of flowering [30,31,32]. *AtARF6* and *AtARF8* redundantly regulate flower maturation and sexual organ development in *Arabidopsis*, and silencing *AqARF6* and *AqARF8* in *Aquilegia* reduces the petal spur length, ultimately decreasing nectar production [33,34,35].

Phytohormones can regulate specific developmental processes synergistically or antagonistically through their deeply interconnected signaling pathways, which allow for the co-regulation of plant development. ARFs are key mediators of auxin response crosstalk with other phytohormones. They establish direct interactions with proteins or cross-regulate genes at the transcriptional level within other signaling pathways, such as ethylene (ETH), brassinosteroid (BR), abscisic acid (ABA), gibberellin (GR), and jasmonic acid (JA), influencing processes like hypocotyl cell elongation, fruit initiation and ripening, and petal growth [36,37,38]. For instance, the BR-regulated kinase Brassinosteroid Insensitive 2 (BIN2) enhances the expression of auxin-induced genes by directly inhibiting *AtARF2*, thereby regulating light-directed seedling growth in *Arabidopsis* [39]. Additionally, *AtARF2* influences the GA signaling pathway by controlling its target genes, thereby regulating greening, flowering time, and senescence in plants [37]. *AtARF6* and its homolog *AtARF8* enhance JA production and play a crucial role in flower development [33]. As a multi-member gene family, the effects of the ARFs involved in flower development regulation on hormone metabolism and signaling remain underexplored.

Petunia (*Petunia hybrida*) is frequently utilized as a model for studying the growth and development of ornamental plants. A developmental map of petunia petals has been established through morphometric measurements at various developmental stages and in different regions of the petal [40]. In this study, 29 ARF proteins were identified in petunia, and 12 highly expressed *PhARFs* were selected from four distinct clades in the phylogenetic tree at two stages of bud development. The full-length cDNAs were isolated, and their biological functions were preliminarily identified through virus-induced gene silencing (VIGS), allowing for the characterization of the phylogenetic features of the *ARFs* involved in flower development. *PhARF5* and *PhARF19a* were identified as non-redundant *ARFs* that significantly impacted corolla morphology and exhibited clear functional differentiation. The impact on differentially expressed genes in the *PhARF5* or *PhARF19a* silencing conditions involved hormone signal transduction, photosynthesis, and various metabolic and biosynthetic processes. Significant differences between *PhARF5-* or *PhARF19a*-silenced petunias in the context of hormone signaling pathways were also discussed.

## 2. Results

### 2.1. Phylogenetic Analysis of Petunia ARF Family Proteins

**Figure 1:**
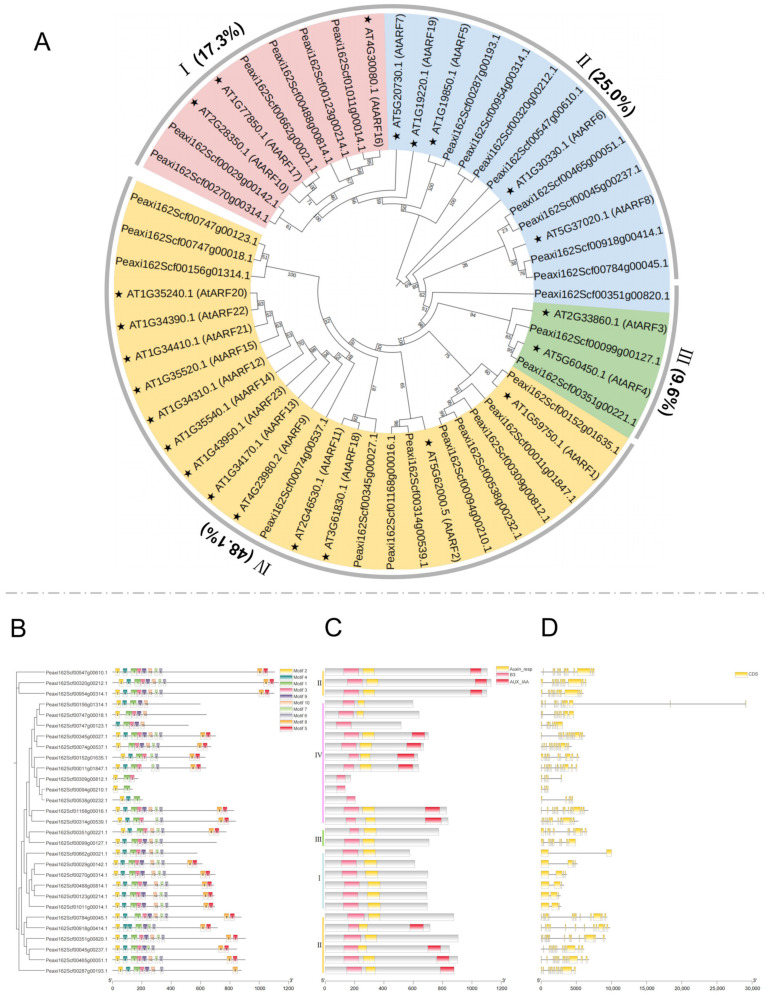
Phylogenetic, gene structural, and motif analyses of ARF proteins. (**A**) Phylogenetic analysis of ARF proteins in *Petunia hybrida* and *Arabidopsis thaliana*. The unrooted tree includes 29 PhARF and 23 AtARF proteins, with stars marking the Arabidopsis ARFs (AtARFs). Branch support values are shown at the nodes. (**B**–**D**) Phylogenetic tree and conserved motifs of PhARF proteins. The unrooted phylogenetic tree was constructed based on the full-length amino acid sequences of 29 PhARF proteins (**B**); Domain organization of PhARF proteins, including the Auxin_resp domain (yellow), B3 domain (pink), and AUX/IAA domain (red) (middle) (**C**); Gene structure of PhARF genes. The exon–intron structures of PhARF genes are shown with yellow boxes representing exons and black lines representing introns (right) (**D**). The length of sequences is indicated by the scale at the bottom.

To elucidate the phylogenetic relationship of the *ARF* gene family in *P. hybrida*, an unrooted phylogenetic tree was constructed using multiple sequence alignment of ARFs from *P. hybrida* in conjunction with 23 ARF proteins from *Arabidopsis* (Figure 1). A total of 52 ARFs was grouped into four major clades: Clade I (six Arabidopsis and three Petunia ARF proteins), clade II (five Arabidopsis and eight Petunia ARF proteins), clade III (two Arabidopsis and three Petunia ARF proteins), and cladeIV (thirteen Arabidopsis and twelve Petunia ARF proteins), which was the largest clade, accounting for 48.1% (Figure 1A) [41]. Utilizing MEME tools, the distribution and structural diversity of conserved motifs among PhARF proteins, which are pivotal for the activation or inhibition of downstream genes through promoter binding, were explored, leading to the exclusion of two PhARFs, Peaxi162Scf00538g00231.1 and Peaxi162Scf00538g00223.1, due to the absence of conserved motifs (Figure 1B) [10]. Ten conserved motifs, each composed of distinct sequences, were identified in 29 ARF proteins of *petunia* (Figure S1). Of these, 11 PhARFs, distributed across different clades, contained all 10 motifs simultaneously (Figure S1). While the other PhARFs did not contain all 10 motifs, the arrangement and composition of the motifs were similar within the same clades (Figure 1B).

Studies have shown that the N-terminal DNA-binding domain (DBD) sequence, classified as a plant-specific B3-type, significantly influences the structure of phylogenetic trees, suggesting functional specificities associated with this domain [42]. All petunia ARF sequences contained a typical N-terminal B3-like DBD (Figure 1B). Further analysis of the conserved domains in PhARF protein sequences revealed that, except for three sequences (Peaxi162Scf00309g00812.1, Peaxi162Scf00094g00210.1, and Peaxi162Scf00538g00232.1), all contained an auxin response domain (Auxin_resp) (Figure 1B). However, the C-terminal domain (CTD) of Aux/IAA, essential for protein–protein interactions, was incomplete in 16 PhARFs, accounting for more than 50% of the sequences (Figure 1B) [11]. Additionally, the gene structure of each *PhARF* was analyzed by comparing the complete coding sequences (CDS) with the corresponding genomic DNA sequences. In *PhARFs*, the number of exons ranged from 2 to 15. The positions and ratios of exons and introns at their respective loci varied greatly, even within the same clade of the *PhARF* gene family (Figure 1).

### 2.2. Broad Clade Distribution of Flower Development-Related PhARFs

**Figure 2:**
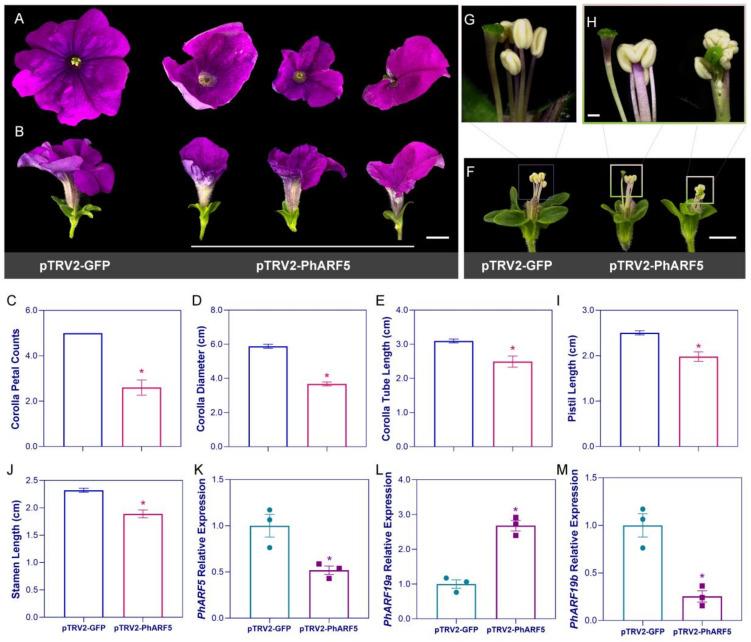
Phenotypic and molecular effects of *PhARF5* silencing on petunia flower development. (**A**,**B**) Representative images of petunia flowers following VIGS-mediated silencing of *PhARF5* compared to pTRV2-GFP controls. Frontal view showing significant reductions in petal number and corolla size in PhARF5-silenced flowers (**A**); side view highlighting the impact on corolla tube length and overall flower structure (**B**). Scale bars represent 1 cm. (**C**–**E**) Quantitative measurements of corolla traits of petal counts (**C**), diameter (**D**), and tube length (**E**) in *PhARF5*-silenced petunias compared to controls. (**F**–**H**) Effects of *PhARF5* silencing on reproductive organ development. Whole flower view (**F**) with scale bars represent 1 cm; close-up views of stamens and pistils from control (**G**) and *PhARF5*-silenced (**H**) flowers with scale bars represent 1 mm. (**I**,**J**) Quantitative measurements of pistil (**I**) and stamen (**J**) lengths in *PhARF5*-silenced petunias compared to controls. (**K**–**M**) Relative expression levels of *PhARF5* (**K**), *PhARF19a* (**L**), and *PhARF19b* (**M**) in *PhARF5*-silenced petunias compared to controls, determined by qPCR. The green dot and purple square represent the expression levels of each biological replicate. Data are presented as mean ± SE; * *p* < 0.05.

**Figure 3:**
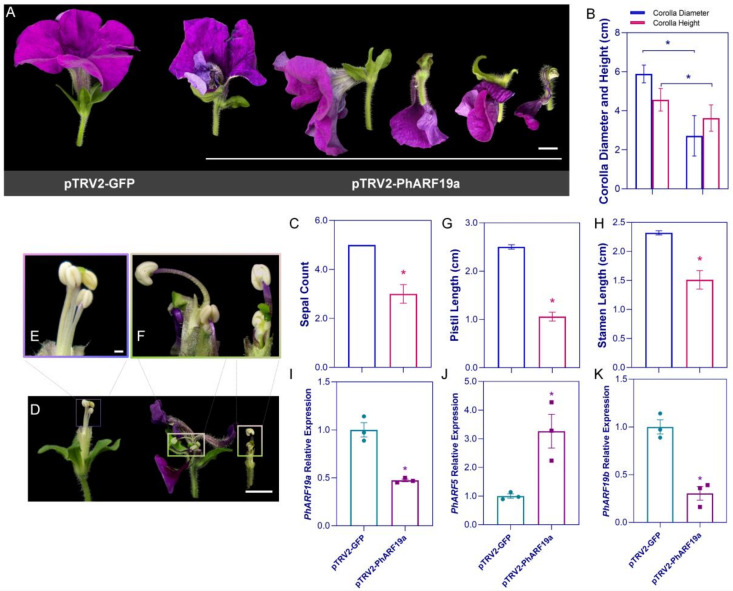
Phenotypic and molecular effects of *PhARF19a* silencing on petunia flower development. (**A**) Representative images of petunia corollas following VIGS-mediated silencing of *PhARF19a* compared to pTRV2-GFP controls. Scale bars represent 1 cm. (**B**,**C**) Quantitative measurements of corolla diameter and height (**B**), sepal count (**C**), in *PhARF19a*-silenced petunias compared to controls. (**D**–**F**) Effects of *PhARF19a* silencing on reproductive organ development. Whole flower view with scale bars represent 1 cm. (**D**). Close-up views of stamens and pistils from control (**E**) and PhARF19a-silenced flowers (**F**) with scale bars represent 1 mm. (**G**,**H**) Quantitative measurements of pistil (**G**) and stamen (**H**) lengths in *PhARF19a*-silenced petunias compared to controls. (**I**–**K**) Relative expression levels of *PhARF19a* (**I**), *PhARF5* (**J**), and *PhARF19b* (**K**) in *PhARF19a*-silenced petunias compared to controls, determined by qPCR. The green dot and purple square represent the expression levels of each biological replicate. Data are presented as mean ± SE; * *p* < 0.05.

We selected 13 highly expressed PhARFs from four distinct clades at two stages of bud development and targeted the silencing of *Peaxi162Scf01011g00014.1* (*PhARF16a*), one of *AtARF16* (AT4G30080.1)’s closer homologs in clade I; silencing *Peaxi162Scf00287g00193.1* (*PhARF5*) and *Peaxi162Scf00320g00212.1* (*PhARF19a*), closer homologs of *AtARF5* (*AT1G19850.1*) and *AtARF19* (*AT1G19220.1*) in clade II, significantly impaired corolla development (Figure 2, Figure 3 and Figure S2, Table S1). In contrast, within this clade, silencing closer homologous genes of *AtARF6/8*, *Peaxi162Scf00465g00051.1* (*PhARF6a*), *Peaxi162Scf00045g00237.1* (*PhARF6b*), and *Peaxi162Scf00784g00045.1* (*PhARF8*), did not lead to any abnormal plant morphology (Figure S3). Additionally, the silencing of members of clade III, including *Peaxi162Scf00099g00127.1* (*PhARF3*), a closer homolog of *AtARF3*, and *Peaxi162Scf00351g00221.1* (*PhARF4*), a closer homolog of *AtARF4*, via VIGS, also resulted in phenotypes that profoundly affected corolla development (Figure S3A–C). In clade IV, specific silencing of *Peaxi162Scf01168g00016.1* (*PhARF2a*), *Peaxi162Scf00074g00537.1* (*PhARF11*), and Peaxi162Scf00345g00027.1 (*PhARF18*), as well as concurrent silencing of *Peaxi162Scf00011g01847.1* (*PhARF1a*) and *Peaxi162Scf00152g01635.1* (*PhARF1b*) by targeting their N-terminal conserved sequences, did not result in abnormal corolla development phenotypes (Figure S3D–K).

### 2.3. PhARF5 Silencing Led to Reduced Petal Number in Gamopetalous Corollas

*PhARF5*-silenced petunias exhibited a predominant phenotype compared to the controls, characterized by a marked reduction in petal number in each corolla (Figure 2A,B). The corollas failed to separate into five limbs, resulting in a 37.5% reduction in the average corolla diameter (Figure 2C,D). Nonetheless, the development of the corolla tube was not significantly impacted, with the average corolla tube length being only slightly reduced by 19.5% (Figure 2E). Beyond influencing the floral organs of the outer whorls, silencing *PhARF5* also impacted the development of sexual organs in *P. hybrida* (Figure 2F). This intervention not only affected the length of pistils and stamens but, more critically, resulted in various deformities (Figure 2G–J). In *PhARF5*-silenced plants, abnormalities such as the fusion of multiple stamens and irregular attachment relationships between pistils and stamens were evident (Figure 2G–J). The overall severity of the floral phenotypes correlated with the extent of *PhARF5* silencing. qPCR analysis indicated that the average relative expression level of PhARF5 in the silenced petunias was reduced by 48.2% compared to the controls (Figure 2K). Furthermore, in the *PhARF5*-silenced petunias, two closely related genes, *PhARF19a* and *PhARF19b* (Peaxi162Scf00954g00314.1), showed contrasting transcriptional changes, with *PhARF19a* being upregulated and *PhARF19b* being downregulated (Figure 2L,M).

### 2.4. PhARF19a Silencing Disrupted Corolla Tube Formation and Upright Corolla Growth

The differences in corolla morphology and positioning between *PhARF19a*-silenced plants and controls were particularly pronounced (Figure 3A). In the controls, the corollas were erect and properly attached to the receptacle, while in *PhARF19a*-silenced petunias, most corollas were tilted or rotated 180 degrees, attaching upside down under the receptacle (Figure 3A). As gamopetalous flowers, *PhARF19a*-silenced petunias failed to form a fully developed corolla tube, with lengths along the *x*-axis and *y*-axis significantly reduced compared to the controls (Figure 3B). The abnormal phenotypic range, besides incomplete corollas, included reduced sepals, often numbering fewer than the typical five (Figure 3C). A comparable phenotype observed in petunias with *PhARF5* silencing was the appearance of deformed pistils and stamens, both exhibiting shorter lengths (Figure 3D–H). In petunia plants with varying degrees of *PhARF19a* silencing, the relative expression of *PhARF19a* decreased by an average of 52.6% (Figure 3I). Concurrently, the relative expression level of its nearby homolog, *PhARF5*, increased, presumably as a compensatory response (Figure 3J). Conversely, the relative expression level of *PhARF19b* consistently showed a significant decline when the abundance of its homologous *PhARFs* was suppressed (Figure 3K).

### 2.5. Sequence Analyses of PhARF5 and PhARF19a in Petunia

The coding regions of *PhARF5* and *PhARF19a* were isolated from petunia ‘Ultra’ and predicted to encode proteins of 879 and 1132 amino acids, with calculated molecular weights of 97.25 kDa and 125.00 kDa, respectively (Figure S4). Phylogenetic analysis was conducted using MEGA 11, constructing trees based on evolutionary distances of ARF5 and ARF19a amino acid sequences from *P. hybrida* and other species, including *S. lycopersicum*, *Nicotiana tabacum*, *Arabidopsis thaliana*, *Vitis vinifera*, *Solanum tuberosum*, *Capsicum annuum*, and *Olea europaea* (Figure S4A). The deduced amino acid sequences of PhARF5 exhibited identities of 87.37% with NtARF5 (XP_016465083.1), 87.18% with CaARF5 (XP_047267293.1), 86.44% with SlARF5 (NP_001234545.1), 86.21% with StARF5 (XP_006342026.1), 68.87% with OeARF5 (XP_022859505.1), 65.93% with VvARF5 (XP_003634382.2), and 57.21% with AtARF5 (NP_001321214.1). Similarly, PhARF19a showed identities of 96% with SlARF19a (NP_001234740.2), 91.67% with CaARF19a (XP_016580257.2), 90.87% with NtARF19a (XP_016515513.1), 90.11% with StARF19a (XP_006365636.1), 76.65% with VvARF19a (XP_010656700.1), 73.07% with OeARF19a (XP_022883057.1), and 72.67% with AtARF19a (NP_173356.1) (Figure S4). These findings suggest that species belonging to the same family, such as petunia, tomato, tobacco, and pepper in the Solanaceae family, display a closer evolutionary kinship, hinting at their shared ancestry and closer evolutionary ties.

Upon examination, the N-terminal sequences of PhARF5 and PhARF19a did not exhibit the bipartite nuclear localization signal (NLS) that is typically observed in most Aux/IAA proteins (Figure S4B) [43,44]. However, analysis revealed nuclear localization signals situated in the middle region (MR) of the sequences. Specifically, the sequence ‘GLKRPFQSAF’ in PhARF5 and ‘FRSKRPRLP’ in PhARF19a were identified as nuclear signals, as illustrated in Figure S4B, suggesting that PhARF5 and PhARF19a proteins may be localized in the nucleus (Figure S4B). In addition to the three conserved domains of B3-like DBD, Auxin_resp, and Aux/IAA, the amino acid sequences of PhARF5 and PhARF19a also feature a glutamate (Q)-rich MR in *P. hybrida* (Figure S4B) [10].

### 2.6. Expression Pattern of PhARF5 and PhARF19a in Petunia

**Figure 4:**
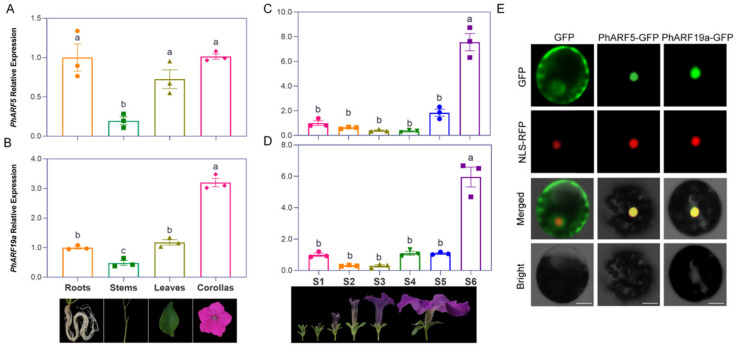
Expression patterns and subcellular localization of *PhARF5* and *PhARF19a* in petunia. (**A**,**B**) Relative expression levels of *PhARF5* (**A**) and *PhARF19a* (**B**) in various petunia organs, including roots, stems, leaves, and corollas, measured by qPCR. The different symbols in the figure represent the expression levels of each biological replicate for different tissues. (**C**,**D**) Expression patterns of *PhARF5* (**C**) and *PhARF19a* (**D**) during different stages of flower development (S1–S6). The different symbols in the figure represent the expression levels of each biological replicate for different stages of flower development. (**E**) Subcellular localization of PhARF5-GFP and PhARF19a-GFP fusion proteins in petunia protoplasts. GFP signals indicate localization, NLS-RFP serves as a nuclear marker, and merged images confirm nuclear localization of both PhARF5 and PhARF19a. Bright-field images provide cell morphology. Scale bars represent 10 µm. Different letters indicate statistically significant differences (*p* < 0.05).

The expression levels of *PhARF5* in various petunia organs were investigated using quantitative RT-PCR (qPCR). High transcript levels were observed in roots, leaves, and corollas, with the highest mean transcript level detected in flowers. However, statistical analysis demonstrated no significant differences in the transcript levels across these tissues (Figure 4A). In contrast, *PhARF19a* exhibited the highest expression level in corollas compared to other organs (Figure 4B). Both *PhARF5* and *PhARF19a* showed the lowest expression levels in stems, indicating a common expression pattern (Figure 4A,B). During flower development, *PhARF5* expression decreased from stage S1 to S4, followed by a continuous increase from S4 to S6, peaking at S6 (Figure 4C). *PhARF19a* showed a similar expression pattern, with an initial decrease from S1, a trough at S3, and a subsequent rise until S6, where it reached its highest level (Figure 4D).

Additionally, our findings indicate that the *PhARF* gene family operated as an interactive unit, as silencing either *PhARF5* or *PhARF19a* affected the transcription of other family members to varying extents. Specifically, in the corolla, Peaxi162Scf00029g00142.1 (*PhARF10a*) from clade I, *PhARF19b* and Peaxi162Scf00547g00610.1 (*PhARF6c*) from clade II, *PhARF11* and *PhARF1b* from clade IV, were all downregulated, regardless of whether PhARF5 or PhARF19a was silenced (Figure S5). Conversely, the loss of one *PhARF* member resulted in the upregulation of specific *PhARFs* from various clades, potentially compensating for the loss of *ARF* function (Figure S5). To examine the subcellular localizations of PhARF5 and PhARF19a, constructs of PhARF5 and PhARF19a fused to GFP were transiently expressed in petunia protoplasts, which coexpressed empty RFP and nRFP (nuclear localization marker). The results demonstrated that both PhARF5 and PhARF19a localized to the nucleus, indicating their nuclear functions (Figure 4E).

### 2.7. Similar Pathway Changes Detected by Transcriptomics in PhARFs-Silenced Petunias

**Figure 5:**
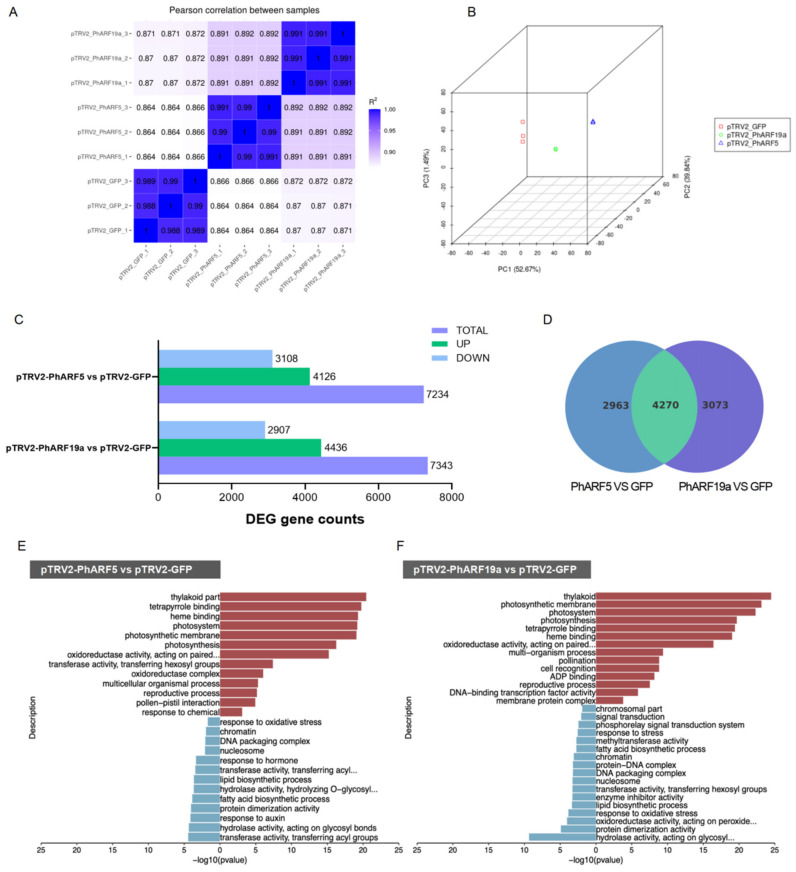
Transcriptome analyses of *PhARF5-* and *PhARF19a*-silenced petunias. (**A**) Pearson correlation heatmap between replicates of pTRV2-GFP (control), pTRV2-PhARF5, and pTRV2-PhARF19a samples. (**B**) Principal component analysis (PCA) of RNA-Seq data from control, PhARF5-silenced, and PhARF19a-silenced petunias. (**C**) Bar chart of differentially expressed genes (DEGs) in *PhARF5*-silenced and *PhARF19a*-silenced petunias compared to the control. (**D**) Venn diagram showing the overlap of DEGs between pTRV2-PhARF5 vs. pTRV2-GFP and pTRV2-PhARF19a vs. pTRV2-GFP. (**E**,**F**) Gene Ontology (GO) enrichment analyses of upregulated (red) and downregulated (blue) DEGs in PhARF5-silenced (**E**) and PhARF19a-silenced (**F**) petunias compared to controls.

RNA sequencing analysis was performed to elucidate the molecular regulatory network characteristics of the *PhARFs* involved in flower development, utilizing RNA libraries constructed from *PhARF5*-silenced, *PhARF19a*-silenced, and control corollas in *P. hybrida*. Pearson’s correlation analysis demonstrated very high correlation values (0.99 or 1) between the replicates of each sample, with the quality control summary of the RNA-Seq data presented in Table S4 and Figure 5A. Principal component analysis (PCA) classified all samples into three distinct groups, indicating significant inter-group differences and good intra-group repeatability of the transcriptomes (Figure 5B). Differentially expressed genes (DEGs) were identified (FDR ≤ 0.05 and |log_2_FC| ≥ 1) through pairwise comparisons of the transcriptomes of *PhARF5*-silenced petunias versus the controls, and *PhARF19a*-silenced petunias versus the controls. In *PhARF5*-silenced petunias, 7234 genes were significantly differentially expressed, with 4126 upregulated and 3108 downregulated compared to the controls (Figure 5C). Similarly, in *PhARF19a*-silenced petunias, 7343 DEGs were identified, with 4436 upregulated and 2907 downregulated compared to the controls (Figure 5C). Venn diagram analysis revealed a substantial overlap of 4270 DEGs between the two comparisons, indicating that their regulatory roles in biological functions may involve many shared pathways (Figure 5D).

To elucidate the functions of *PhARF5* in petunia, we conducted Gene Ontology (GO) and Kyoto Encyclopedia of Genes and Genomes (KEGG) pathway analyses (Figure 5E and Figure S6A and Tables S5 and S6). GO analysis of the upregulated genes in *PhARF5*-silenced petunias revealed enhancements in the photosynthesis-related processes, including ‘Thylakoid part’, ‘Photosynthetic membrane’, ‘Photosystem’, and ‘Photosynthesis’ (Figure 5E, Table S5). Molecular function analysis showed enrichment in ‘Tetrapyrrole binding’ and ‘Heme binding’, essential for chlorophyll II synthesis, and ‘Oxidoreductase activity’, linked to enhanced redox reactions, suggesting increased photosynthetic activity (Figure 5E, Table S5). Additionally, terms like ‘Transferase activity, transferring hexosyl groups’ and ‘Multicellular organismal process’ indicated changes in carbohydrate metabolism and broader developmental processes, consistent with the KEGG analysis, showing shifts in the primary metabolic processes, such as ‘Carbon metabolism’, ‘Nitrogen metabolism’, and ‘Glycine, serine, and threonine metabolism’, to support increased energetic and biosynthetic demands (Figure 5E and Figure S6A). The KEGG analysis also highlighted enrichment in ‘Zeatin biosynthesis’ and ‘Plant hormone signal transduction’, suggesting significant changes in hormonal balance and signaling, particularly cytokinins, crucial for cell division and growth (Figure S6A). Upregulated DEGs were also enriched in the ‘Reproductive process’ and ‘Pollen–pistil interaction’, indicating potential alterations in reproductive mechanisms (Figure 5E). Conversely, the GO analysis of downregulated genes revealed suppression of stress and hormone responses, particularly ‘Response to oxidative stress’, suggesting reduced activity in the stress response pathways, and the ‘Response to hormone’, particularly auxin, critical for various growth and developmental processes (Figure 5E, Table S6). The downregulation of ‘nucleosome’ and ‘chromosome’ components indicated potential impacts on the chromatin structure and gene regulation, supported by the KEGG analysis of enrichment in ‘Protein dimerization activity’ and ‘DNA packaging complex’ (Figure 5E and Figure S6B). Moreover, the significant reduction in ‘transferase and hydrolase activities’ suggested broader downregulation of metabolic enzyme functions, such as ‘Lipid biosynthetic process’ and ‘Fatty acid biosynthetic process’, consistent with the KEGG analysis results (Figure 5E and Figure S6B, Table S6).

The GO analysis of upregulated DEGs in *PhARF19a*-silenced petunia also revealed significant enrichment in the photosynthesis-related processes, including terms such as ‘Thylakoid’, ‘Photosynthetic membrane’, ‘Photosystem’, ‘Photosynthesis’, ‘Tetrapyrrole binding’, and ‘Heme binding’ (Figure 5F, Table S7). This indicates that silencing *PhARF19a* may enhance photosynthetic activity, potentially as a compensatory response to stress or altered metabolic demands. Furthermore, the enrichment of biological processes such as ‘Multi-organism process’, ‘Pollination’, ‘Reproductive process’, and ‘Pollen recognition’ suggests profound changes in the developmental and reproductive mechanisms, which could impact fertility and flower development (Figure 5F, Table S7). The involvement of pathways related to ‘DNA-binding transcription factor activity’ and ‘Membrane protein complex’ underscored the broader impacts on gene regulation and membrane protein functions, thereby influencing various cellular activities (Figure 5F, Table S7). Additionally, the KEGG analysis reinforced the role of *PhARF19a* in hormone regulation, showing notable enrichment in ‘Plant hormone signal transduction’ and ‘Zeatin biosynthesis’, similar to *PhARF5*-silenced petunias, highlighting enhanced cytokinin synthesis (Figure S6C). The increased expression of some genes involved in ‘Phenylpropanoid biosynthesis’ implied a potential augmentation in antioxidant capacity and the structural integrity of the plant (Figure S6C). Conversely, downregulated genes exhibited suppression in lipid biosynthetic processes, including the ‘lipid biosynthetic process’ and ‘fatty acid biosynthetic process’ (Figure 5F, Table S8). The KEGG pathway analysis confirmed this suppression, showing reduced activity in the primary and secondary metabolic pathways, such as ‘amino sugar and nucleotide sugar metabolism’, ‘fatty acid biosynthesis’, ‘fatty acid elongation’, and ‘glycerolipid metabolism’ (Figure S6D). The diminished activity in ‘Response to stress’ and ‘Response to oxidative stress’ suggested altered stress management mechanisms, further supported by the reduced transcription of genes involved in ‘Phenylalanine’, ‘tyrosine’, and ‘tryptophan biosynthesis’ (Figure 5F and Figure S6D and Table S8). Moreover, downregulation in the ‘Signal transduction’ and ‘Phosphorelay signal transduction system’ categories indicated decreased activity in the signal transduction pathways, potentially linked to hormonal signaling disruptions (Figure 5F and Figure S6D and Table S8).

In summary, enrichment analyses of *PhARF5-* and *PhARF19a*-silenced petunias both revealed enhanced photosynthetic activity, modifications in global hormonal pathways, and regulation of nuclear components. They influenced central carbon and nitrogen metabolism and secondary metabolite biosynthesis, underscoring their roles in metabolic and biosynthetic processes. Additionally, they played a crucial role in managing oxidative stress, highlighting their involvement in stress and redox responses. Furthermore, they impacted reproductive mechanisms, affecting fertility and flower development.

### 2.8. Profound Shifts in Endogenous Hormone Levels Detected in PhARFs-Silenced Corollas

**Figure 6:**
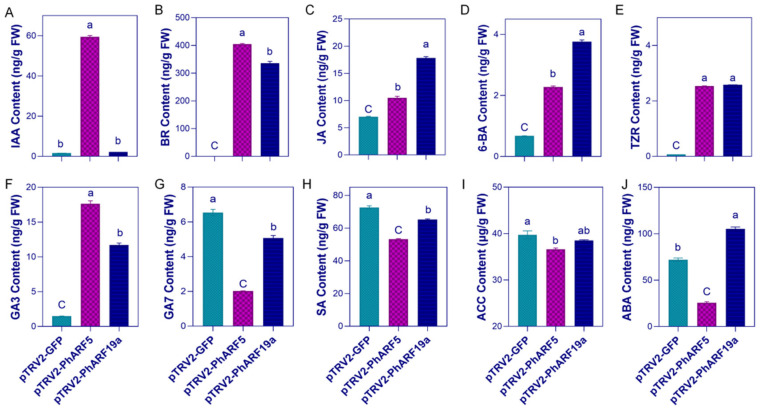
Phytohormone content analyses in *PhARF5-* and *PhARF19a*-silenced petunias. (**A**–**J**) Quantification of phytohormone levels of indole-3-acetic acid (IAA) (**A**); brassinosteroid (BR) (**B**); jasmonic acid (JA) (**C**); 6-Benzylaminopurine (6-BA) (**D**); Trans-Zeatin-riboside (TZR) (**E**); gibberellin 3 (GA3) (**F**); gibberellin 7 (GA7) (**G**); salicylic acid (SA) (**H**); 1-Aminocyclopropane-1-carboxylic acid (ACC) (**I**); abscisic acid (ABA) (**J**), in controls, *PhARF5*, and *PhARF19a*-silenced petunias using UPLC-MS/MS. Different letters indicate statistically significant differences (*p* < 0.05).

To explore whether silencing *PhARFs* disrupts auxin signaling, which in turn induces crosstalk with other hormone signals, thereby contributing to flower development, we quantified the levels of common phytohormones using UPLC-MS/MS. In plants with reduced *PhARF5* mRNA levels, Auxin, typified by indole-3-acetic acid (IAA), was elevated approximately 37-fold compared to the controls. In contrast, *PhARF19a*-silenced plants did not exhibit a significant feedback response in auxin signaling (Figure 6A). The trends in changes of the most endogenous plant hormones were consistent when either *PhARF5* or *PhARF19a* was silenced, although the extent of the changes varied. Hormones such as brassinosteroid (BR), jasmonate (JA), the cytokinins (CTKs) 6-BA (6-Benzylaminopurine) and TZR (Trans-Zeatin-riboside), and gibberellin 3 (GA3) were significantly upregulated. Conversely, gibberellin 7 (GA7) and salicylic acid (SA) were downregulated (Figure 6B–H). The total level of 1-aminocyclopropane-1-carboxylic acid (ACC), a substrate for ethylene (ETH) synthesis, was very high in the petunia corolla, and silencing *PhARF5* led to a reduction in its level compared to the control, but no significant difference was observed compared to the *PhARF19a*-silenced petunias (Figure 6I). However, abscisic acid (ABA) exhibited the opposite trend, as it was significantly downregulated in petunia following *PhARF5* silencing, whereas *PhARF19a* silencing led to a significant increase in its content (Figure 6J).

### 2.9. Differential Regulation of PhARFs in Hormone Signaling Pathways

**Figure 7:**
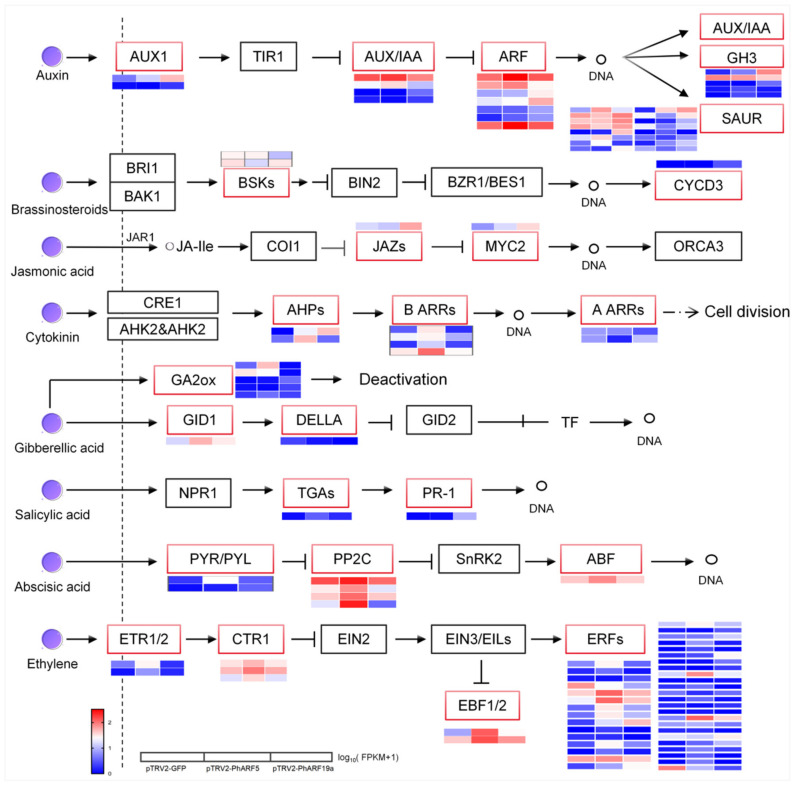
Differential expression of hormone signaling pathway genes in PhARF5- and PhARF19a-silenced petunias. Heatmap representation of differentially expressed genes (DEGs) in various hormone signaling pathways of auxin, brassinosteroid, jasmonic acid, cytokinin, gibberellic acid, salicylic acid, abscisic acid, ethylene in pTRV2-GFP (control), pTRV2-PhARF5, and pTRV2-PhARF19a-silenced petunias. Auxin signaling pathway: AUX1 (Auxin Resistant 1), TIR1 (Transport Inhibitor Response 1), AUX/IAA (Auxin/Indole-3-Acetic Acid), ARF (auxin response factor), GH3 (Gretchen Hagen 3), and SAUR (Small Auxin-Up RNA); Brassinosteroid signaling pathway: BRI1 (Brassinosteroid Insensitive 1), BAK1 (BRI1-Associated Receptor Kinase 1), BSKs (Brassinosteroid-Signaling Kinases), BIN2 (Brassinosteroid Insensitive 2), BZR1/BES1 (Brassinazole Resistant 1/BRI1-EMS-Suppressor 1), CYCD3 (Cyclin D3); Jasmonic Acid Signaling Pathway: COI1 (Coronatine Insensitive 1), JAZs (Jasmonate ZIM-Domain Proteins), and MYC2 (Myelocytomatosis 2); Cytokinin Signaling Pathway: CRE1 (Cytokinin Response 1), AHK2 and AHK3 (Histidine Kinase 2 and 3), AHPs (Histidine-Containing Phosphotransfer Proteins), B-ARRs (Type-B Response Regulators), and A-ARRs (Type-A Response Regulators); Gibberellic Acid Signaling Pathway: GA2ox (Gibberellin 2-Oxidase), GID1 (Gibberellin Insensitive Dwarf 1), DELLA (DELLA Protein), and GID2 (Gibberellin Insensitive Dwarf 2); Salicylic Acid Signaling Pathway: NPR1 (Nonexpresser of Pathogenesis-Related Genes 1), TGAs (TGA Transcription Factors), and PR-1 (Pathogenesis-Related Protein 1); Abscisic Acid Signaling Pathway: PYR/PYL (Pyrabactin Resistance/Pyrabactin Resistance-Like), PP2C (Protein Phosphatase 2C), SnRK2 (Snf1-Related Protein Kinase 2), and ABF (Abscisic Acid-Responsive Element-Binding Factor); Ethylene Signaling Pathway: ETR1/2 (Ethylene Receptor 1/2), CTR1 (Constitutive Triple Response 1), EIN2 (Ethylene Insensitive 2), EIN3/EILs (Ethylene Insensitive 3/Ethylene Insensitive-Like), ERFs (Ethylene Response Factors), and EBF1/2 (EIN3-Binding F-Box 1/2).

Although the transcriptional effects of homologs *PhARF5* and *PhARF19a* were similar compared to the control, a total of 5631 DEGs (3006 upregulated and 2625 downregulated) were detected in the comparison between *PhARF5*- and *PhARF19a*-silenced petunias, with approximately 87.5% of these DEGs overlapping with those found in the PhARF5-silenced or PhARF19a-silenced vs. control groups. This suggests that notable differences in similar pathways were caused by different *PhARFs* involved in flower development (Figure S7A,B). Given the functional differentiation between *PhARF5* and *PhARF19a* and the significant enrichment of DEGs in plant hormone signal transduction through the GO and KEGG analyses of *PhARF5-* and *PhARF19a*-silenced petunias, as well as pairwise comparisons of the silenced *PhARFs* and controls, we identified DEGs in the hormone signaling pathways to deepen our understanding of *PhARFs*’ regulatory roles during the morphological changes in flower development (Figure S7, Table S9). In the auxin signaling pathway, 34 genes were differentially expressed between *PhARF5-* and *PhARF19a*-silenced petunias, indicating the distinct regulatory role of auxin signaling in corolla morphology. Among these, seven DEGs were identified as *ARFs*, two as *AUX1/LAXs*, four as *AUX/IAAs*, sixteen as *SAURs*, and five as *GH3s* (Figure 7, Table S10). Compared to the control, most of these DEGs (22/34 in *PhARF5*-silenced and 25/34 in *PhARF19a*-silenced plants) were upregulated in *PhARF*-silenced petunias. Additionally, these genes were expressed at higher levels in the *PhARF19a*-silenced petunias (22/34) compared to the *PhARF5*-silenced petunias (Figure 7, Table S10).

In the BR signaling pathways, two DEGs encoding BR-signaling kinase (BSKs) and one DEG encoding Cyclin D3 (CYCD3), reported to be a downstream gene influenced by BRs in cell proliferation, were identified (Figure 7, Table S10) [45]. Additionally, in the JA signaling pathways, one DEG encoding Jasmonate ZIM-domain (JAZ) protein and one JAZ-repressed *Myelocytomatosis 2* (*MYC2*) in the JA signaling pathway were found (Figure 7, Table S10). No obvious pattern of upregulation or downregulation was observed, suggesting comprehensive regulation of these pathways. In the CTK signaling pathways, eight DEGs were identified, including two encoding histidine-containing phosphotransfer proteins (AHPs), four type-B response regulators (B-ARRs), and two type-A response regulators (A-ARRs). Most of these DEGs were upregulated in *PhARF5*-silenced plants compared to the *PhARF19a*-silenced plants or controls, while they were downregulated in the *PhARF19a*-silenced plants compared to the controls (Figure 7, Table S10). Only two DEGs were identified in the GA signaling pathway: one encoding Gibberellin Insensitive Dwarf 1 (GID1) and the other encoding its possible interacting DELLA protein, RGA-Like 2 (RGL2). However, five genes encoding Gibberellin 2-oxidase (GA2ox) enzymes, which catalyze the conversion of active GAs into their inactive forms, were differentially expressed between the *PhARF5*-silenced and *PhARF19a*-silenced plants, indicating various roles of *PhARFs* in maintaining the balance of active GAs (Figure 7, Table S10) [46]. In the SA signaling pathway, two DEGs, one encoding a TGA transcription factor (TGA) and the other as the TGA’s possible target gene, encoding Pathogenesis-Related Protein 1 (PR-1), were identified between the *PhARF5*-silenced and *PhARF19a*-silenced plants, both of which were upregulated compared to the controls (Figure 7, Table S10). In the ABA signaling pathway, seven DEGs were identified between the *PhARF5*-silenced and *PhARF19a*-silenced plants, including two from the Abscisic Acid Receptor PYR/PYL families (PYLs), four encoding Protein Phosphatase 2C, and one ABA-responsive element binding factor (ABF). Most of these genes were significantly upregulated in the *PhARF5*-silenced petunias compared to the *PhARF19a*-silenced petunias and the controls (Figure 7, Table S10). Silencing *PhARF19a* reduced the expression of these genes compared to the controls, except for one of the *PYLs*, which was significantly higher than in the controls, and *PhARF5*-silenced petunias, as well (Figure 7, Table S10). In petunia plants where different *PhARF* genes were silenced, we found many DEGs in the ethylene signaling pathway, including 46 in total, with a significant number of Ethylene-responsive transcription factors (ERFs) (39), as well as two Ethylene Receptor 2 (ETR2), three Constitutive Triple Response 1 (CTR1), and two EIN3-Binding F-BOX Protein 2 (EBF2) (Figure 7, Table S10).

## 3. Discussion

Flower morphology influences the interactions with pollinators, predators, and various abiotic environmental factors, playing a crucial role in plant evolutionary adaptation [47]. In this study, we identified auxin-responsive factors (PhARFs) in petunia that regulate early auxin-responsive genes’ transcription within the auxin signaling pathway and characterized their roles in flower development. *PhARF5* and *PhARF19a*, two non-redundant *PhARFs*, were further examined to explore their commonalities and differences in transcriptional regulation related to flower development.

### 3.1. Members of the ARF Family Have Revealed the Evolution of P. hybrida

The ARF family has been extensively identified in the genomes of various species, including 15 in *Ginkgo biloba* L, 23 in *A. thaliana*, 25 in *O. sativa*, 21 in *S. lycopersicum*, 22 in *C. annuum*, and 46 in *N. tabacum*. In this study, we identified 29 PhARFs containing the N-terminal DBD in the genome of *Petunia hybrida* (Figure 1) [41,48,49,50,51]. A phylogenetic tree was constructed to analyze the relationships within the ARF family between *A. thaliana* and *P. hybrida*. This family was divided into four major clades: clade I contained six PhARFs and three *Arabidopsis* homologs (AtARF10/16/17); clade II included eight PhARFs and five *Arabidopsis* homologs (AtARF5/6/7/8/19); clade III comprised three PhARFs and two *Arabidopsis* homologs (AtARF3/4); and clade IV encompassed twelve PhARFs and thirteen *Arabidopsis* homologs (AtARF1/2/9/11/12/13/14/15/18/20/21/22/23) (Figure 1). These findings are consistent with previous studies in other species [41,51,52]. Notably, clades I to III all had more duplicated *ARFs* in the genome of petunia than in *Arabidopsis*, suggesting that petunia may have undergone more diverse selection during its evolution [53]. Ten conserved motifs were identified in *PhARFs*, indicating that common or specific motifs are differentially present across various *PhARFs* from different clades through phylogenetic analysis (Figure 1 and Figure S1). Although the number of motif members varied among clades, the same clade motif patterns were relatively conserved, suggesting that *PhARFs* within the same class may have redundant functions, similar to the redundancy observed in *AtARF7* and *AtARF19* in clade II of *Arabidopsis* [16]. This aligns with the increased duplication events in petunia, which may reduce selective constraints [54]. The number of exons in *PhARFs* ranged from 2 to 15 (Figure 1), consistent with previous reports in species such as *Zea mays* and *Carica papaya* [55,56], suggesting that the gene structures of *ARF* family members in plants exhibit significant diversity, which may be related to their various regulatory functions [57].

### 3.2. The Phylogenetic Profiles of ARFs Implicated in Flower Development Lack Distinct Clade-Specific Patterns

Here, thirteen *PhARFs* from four distinct clades exhibiting high expression levels at two stages of floral bud development were silenced using the VIGS method. Silencing *PhARF16a* of clade I, *PhARF5* and *PhARF19a* of clade II, and *PhARF3* and *PhARF4* of clade III led to notable alterations in petunia corolla morphology, including limb splitting, the emergence of additional petals, a reduction in petal number, and abnormalities in the positioning and development of the corolla tube (Figure 2, Figure 3, Figures S2 and S3). We silenced *PhARF1a*, *PhARF1b*, *PhARF11*, and *PhARF18* in clade IV and observed no effect on corolla morphology (Figure S3, Table S1). However, *Arabidopsis arf2* mutants in this clade displayed flowers with altered morphology and long, thick, inflorescence stems; thus, it cannot be ruled out that the other homolog of AtARF2 (AT5G62000.5) in clade IV, *Peaxi162Scf00314g00539.1* or *Peaxi162Scf01168g00016.1*, may play a regulatory role in floral development [31]. The function of clade IV has been reported in rice, where *OsARF11* affects leaf development and participates in stress responses, and *OsARF1* influences leaf size [22,26,58]. Our results suggest that *ARFs* involved in the regulation of flower development are not clade-specific but are widely distributed across various clades, participating in the regulation of specific floral traits. This is unlike the *MYB* gene family related to anthocyanin accumulation, which is mainly concentrated in subgroup 6 (SG6) and contains an R2 and an R3 repeat [59]. All *PhARFs* related to flower development contained the typical DBD (B3-like) and an Auxin_resp domain (Figure 1 and Figure S3), functioning as transcription factors (TFs) to mediate ARF-activated gene expression [42]. Additionally, *PhARF5* and *PhARF19a* also contained a C-terminal domain (CTD) (Figure 1), suggesting that these proteins may interact with Aux/IAA or other ARFs to synergistically or antagonistically regulate flower development [11]. Previous studies have reported that *Arabidopsis arf6 arf8* double-null mutant flowers were arrested as infertile closed buds with short petals and short stamen filaments [33]. This indicates that homologous *AtARF6* and *AtARF8* redundantly regulate flower development. Our findings are consistent with this, as silencing *PhARF6a*, *PhARF6b*, and *PhARF8* alone resulted in no flower morphological changes (Figure S3D–K). However, significant flower morphological changes were observed when silencing the homologous pair *PhARF5* and *PhARF19a* and *PhARF3* and *PhARF4* alone, which is inconsistent with the redundancy observed in *AtARF6* and *AtARF8*. In *Arabidopsis*, *AtARF3* and *AtARF5* are individually involved in regulating flower development [28,30].

### 3.3. PhARF5 and PhARF19a Regulate Distinct Ornamental Traits of the Petunia Corolla

Further investigation into the functions of the homologous pairs *PhARF5* and *PhARF19a* revealed that the floral phenotype of *PhARF5*-silenced petunia was similar to that of the *Arabidopsis mp* (*arf5*) mutant, which exhibited flowers with short and reduced petals, short stamen filaments, and immature gynoecia (Figure 2) [28]. This suggests that the inability of limbs to separate normally in petunia may be due to *PhARF5*’s role as *MPs*, essential for organ primordium initiation [60]. Consistent with the phenotype observed in *PhARF5*-silenced petunias, the abnormal sexual organ morphology in *PhARF19a*-silenced petunias suggests that *PhARF5* and *PhARF19a* may exhibit partial functional redundancy in regulating sexual organ development. Compensatory transcription was observed between the closely related genes *PhARF5* and *PhARF19a* when either was silenced (Figure 2 and Figure 3). However, the abnormal development of the corolla tube and the orientation of the corolla on the receptacle in *PhARF19a*-silenced petunias, which has not been previously reported, indicate that neither *PhARF5* nor *PhARF19a* can fully compensate for the loss of the other (Figure 2 and Figure 3). This is in contrast to the redundancy observed between *AtARF1* and *AtARF2* in senescing leaves [61]. Existing studies in *O. sativa* and *Arabidopsis* have shown that *ARF19* influenced leaf cell expansion and leaf size [18,19,20]. And *OsARF19* and *SlARF19* were induced by abiotic stress [22,23,24]. The transcription of another homolog, *PhARF19b*, was downregulated both in *PhARF5-* or *PhARF19a*-silenced petunias (Figure 2 and Figure 3), suggesting that it may be induced by *PhARF5* or *PhARF19a* as an enhancer rather than as a core TF regulating flower development in petunias. PhARF5 and PhARF19a exhibited high identity with ARF5 and ARF19 in other plants and share the same Q-rich MR, consistent with other species (Figure S4) [62], which suggests that they function as transcriptional activators of downstream genes, aligning with their positive regulation of flower morphology (Figure 2 and Figure S4).

### 3.4. Functional Divergence Induced Within Analogous Pathways by Homologous ARFs

*PhARF5* and *PhARF19a* are both involved in regulating flower development, and our results demonstrate that they exhibit similar expression patterns across various tissues and stages of flower development. This similarity is consistent with the expression patterns of *AtARF5* and *AtARF19* in *Arabidopsis*, where they are strongly expressed in roots, leaves, and flowers, although the role of *AtARF19* in flowers remains unknown [17,18,25]. Furthermore, expression patterns at different stages of flower development in petunia revealed that in addition to their high transcript abundance during early floral bud development (Tables S1 and S2), *PhARF5* and *PhARF19a* expression peaks at S5 (anthesis), indicating their critical role in flower opening (Figure 4C,D). As expected, PhARF5 and PhARF19a proteins were both localized to the nucleus (Figure 4E), reinforcing the notion that ARFs are among the three essential components (TIR1/AFBs, Aux/IAAs, and ARFs) for nuclear auxin signaling [63].

Despite *PhARF5* and *PhARF19a* regulating different ornamental traits of the petunia corolla, transcriptome analysis revealed a significant overlap in DEGs between *PhARF5-* and *PhARF19a*-silenced petunias compared to the controls. This overlap indicates that these closely related *ARFs* influence analogous pathways. However, within these shared pathways, functional divergence occurs, as evidenced by the distinct effects of gene silencing. Upregulated DEGs were involved in photosynthesis, nuclear events, and hormone signal transduction, while downregulated DEGs were linked to stress responses, hormone responses, and fatty acid biosynthesis. DEGs related to hormone metabolism, central carbon and nitrogen metabolism, and secondary metabolite biosynthesis exhibited both upregulation and downregulation (Figure 5 and Figure S6, Tables S5 and S6). These patterns closely resemble the regulatory pathways of *SlARF6A* in *S. lycopersicum* fruit ripening and *MsARFs* in *Medicago sativa* stress responses, suggesting a relatively conserved downstream regulatory network of *ARFs* with differential functions [41,64]. The observed changes in hormone metabolism in these silenced petunias further support this functional divergence within shared pathways (Figure 6). Moreover, *PhARF5* and *PhARF19a* similarly influence the levels of BR, JA, 6-BA, TZR, GAs, SA, and ACC, promoting or inhibiting them to varying extents. *AtARF6* and its close homolog *AtARF8* promoted JA production and played a crucial role in flower development, underscoring the importance of JA in this process [33]. Our results suggested that, beyond JA, *PhARF5* and *PhARF19a* regulate flower development through broader hormonal changes, indicating their involvement in complex feedback mechanisms with other phytohormones (Figure 6). Additionally, the upregulated DEGs were specifically enriched in pathways related to pollination and pollen recognition, likely due to the influence of *PhARF5* and *PhARF19a* on sexual organ development (Figure 2F–H and Figure 3D–H). The DEGs related to overall metabolism, along with significantly enhanced photosynthetic and oxidoreductase activities, may reflect an increased energy demand as a compensatory response to the physiological stress triggered by the absence of *PhARF* transcription [65,66].

### 3.5. Homologous ARFs Exert Markedly Different Effects on Analogous Pathways

The transcriptomic analysis revealed that while *PhARF5* and *PhARF19a* influenced analogous pathways, silencing either gene resulted in differential effects on specific *PhARFs*. These differences likely contribute to the significant morphological changes observed in the flowers of *PhARF5*- and *PhARF19a*-silenced petunias (Figure 2, Figure 3, and Figure S5). This pattern aligns with the mechanisms observed in other species, where multiple *ARFs* are activated to respond to stress conditions [24]. Silencing *PhARF5* in petunia led to a substantial increase in endogenous auxin levels, suggesting that *PhARF5* exerts negative feedback on auxin, likely due to the *MP* gene’s involvement in auxin-based feedback control [67]. In contrast, this feedback on auxin metabolism did not occur in *PhARF19a*-silenced petunias. Additionally, *PhARF5* and *PhARF19a* had opposing effects on the feedback regulation of ABA metabolism. Such contrasting effects are consistent with the detection of 5631 DEGs when comparing the two silenced groups, further supporting the functional divergence of *PhARF5* and *PhARF19a*. The transcriptomic analysis also showed that *PhARF5* and *PhARF19a* significantly influenced plant hormone signal transduction and metabolism, which was also the main pathway enriched in DEGs in the comparison group of *PhARF5-* and *PhARF19a*-silenced petunias. In other plants, most *ARFs* regulate growth and development by modulating auxin signaling, engaging in crosstalk with other phytohormones, or affecting phytohormone metabolism [31,33,34,36,60]. For example, in *Arabidopsis,* MP interacts with Dornroschen-like (DRNL), an AP2-type TF, to form a protein complex that directly activates *AHP6* and *Cytokinin Oxidase/Dehydrogenase 6* (*CKX6*), inhibiting cytokinin signaling and promoting its degradation to synergistically limit cytokinin signals in the primordium initiation area, thereby enabling lateral organ initiation [60]. Therefore, in petunia, the abnormal separation of the petunia limb in *PhARF5*-silenced plants is likely due to downregulated *PhARF5* transcription, which may interfere with the cytokinin signaling pathway and affect the formation of flowers (a type of lateral organ). Five genes encoding two AHPs and three B-ARRs in the cytokinin signaling pathway were significantly upregulated compared to the control, with distinct differences observed in *PhARF19a*-silenced petunias (Figure 7, Table S10), partially explaining the different phenotype seen in *PhARF19a*-silenced plants. *AtARF19* and *AtARF7*, along with ethylene biosynthesis and signaling, are required for PABA to regulate the asymmetric auxin response and local auxin biosynthesis, thereby facilitating the root gravity response [68]. Additionally, OsARF19 controls rice leaf angles by positively regulating *OsGH3-5* and *Brassinosteroid Insensitive 1* (*OsBRI1*) [21]. We examined the effect of *PhARF19a* on brassinosteroid biosynthesis and found that two *PhBSKs* in the brassinosteroid signaling pathway were significantly inhibited compared to the control and distinctly different from those in *PhARF5*-silenced petunias. Concurrently, the expression of the downstream target gene *CYCD3*, associated with cell proliferation and cytokinin responses, was upregulated in *PhARF19a*-silenced petunias. Furthermore, *PhARF19a* did not affect the biosynthesis of the ethylene precursor ACC but broadly impacted the ethylene signaling pathway (Figure 7, Table S10). *PhARF5* and *PhARF19a*, which regulated flower development, influenced a wide range of phytohormone biosynthesis and signaling pathways. However, their most direct impact lies in modulating the auxin signaling pathway, which in turn triggered signal crosstalk directly or indirectly [6,57]. This suggests that *PhARF5* and *PhARF19a* primarily influence plant growth and development through the regulation of auxin signaling, a process that extends beyond auxin itself, involving complex interactions between auxin and other plant hormone signals. These direct and indirect interactions work together to coordinate the intricate processes of plant growth and development.

## 4. Materials and Methods

### 4.1. Plant Materials and Growth Conditions

Petunia ‘Ultra’ seeds were purchased from Sanli Horticulture Co., Ltd., and all materials were cultivated in climate chambers (22–25 ℃, 14 h light/10 h dark, 60% relative humidity) at Zhejiang Agriculture and Forestry University (Hangzhou, China).

### 4.2. Sequence Analysis, Phylogenetic Tree Construction, and Motif Composition of the PhARFs

The amino acid sequences of *A. thaliana* were obtained from TAIR (https://www.arabidopsis.org/ (accessed on 3 June 2024)), while sequences from other species were retrieved from the NCBI BLAST network server (https://blast.ncbi.nlm.nih.gov/Blast.cgi (accessed on 7 June 2024)). Multiple sequence alignment was performed using MUSCLE Wrapper implemented in TBtools (version 2.119, China), followed by trimming using TrimAL Wrapper [69]. The maximum likelihood phylogenetic tree was constructed using IQ-TREE version 1.6.12, with the JTT + R6 model identified as the best-fit sub-phylogenetic model by ModelFinder, and Ultrafast bootstrap analysis was set to 100 [70,71]. Based on evolutionary relationships, the PhARF and AtARF proteins were classified into clades I-IV. The MEME 4.12.0 online tool (https://meme-suite.org/meme/ (accessed on 3 June 2024)) was used to identify the conserved motifs in the 29 PhARF proteins, and the motif features were visualized with TBtools [69].

### 4.3. Agroinoculation of pTRV2 Vectors

To investigate whether the *ARFs* regulating corolla formation in petunia form a single monophyletic clade with high bootstrap support, akin to many genes involved in anthocyanin biosynthesis [72,73], and given that the amino acid sequences of PhARFs show low sequence homology with ARFs in *A. thaliana*, complicating functional annotation, we selected 13 highly expressed *PhARFs* at two stages of bud development from four distinct clades based on the transcriptome analyses reported by Guo et al. (2017) [74]. To silence the corresponding genes via the virus-induced gene silencing (VIGS) system, the primers were designed using the Petunia axillaris v 1.6.2 genome from the Sol Genomics Network (https://www.sgn.cornell.edu/tools/blast/?db_id=272 (accessed on 3 June 2024)) to amplify 276 bp, 426 bp, and other fragments from the 3′ untranslated regions (UTRs) of *PhARF5*, *PhARF19a*, and other *PhARFs*. The VIGS primers used for amplification are detailed in Table S1. For virus-induced gene silencing, the pTRV1 vector was used alongside pTRV2 in the VIGS system, and Table S1 specifies the amplified gene and corresponding region for each primer. These fragments were then individually inserted into the pTRV2 vector. *Agrobacterium tumefaciens* transformed with pTRV2 derivatives and pTRV1 vectors were prepared following previously established methods [75]. pTRV2-derivative vectors included pTRV2-35S::GFP as a control and pTRV2-35S::X, where ‘*X*’ represents one of the twelve *PhARFs* with fragment lengths ranging from 250 to 550 bp. Thirty petunia plants were inoculated with each vector and grown under greenhouse conditions (22–25 °C, 14 h light/10 h dark, 60% relative humidity). Three biological replicates corresponding to three independent positive transformation events were included in these analyses.

### 4.4. Quantitative Real-Time PCR Assays

Quantitative real-time PCR (qPCR) was performed following the protocol outlined by Liu et al. [76]. The analyses adhered to the *Minimum Information for Publication of qPCR Experiments* (MIQE) guidelines [77]. *Cyclophilin* (*CYP*) (accession No. EST883944) served as the internal reference gene. Data analysis was carried out using the method described by Livak and Schmittgen [78]. The sequences employed in the qPCR assay, designed to amplify *PhARF5* and *PhARF19a*, are listed in Table S2. This experiment was conducted to quantify the expression levels of *PhARF5* and *PhARF19a* to assess their roles in regulating flower development. All experiments were conducted in triplicate with independently collected and extracted biological samples.

### 4.5. Subcellular Localization

The coding sequences of PhARF5 and PhARF19a were amplified using specific primers (Table S3) based on sequences from the petunia genome database (https://solgenomics.net/organism/Petunia_axillaris/genome (accessed on 15 October 2023)). The amplified fragments were then cloned into the pSAT-1403TZ vector, where the GFP expression is driven by the CaMV 35S promoter. Petunia leaf protoplasts were isolated and transfected with plasmids using polyethylene glycol-mediated transformation, following the protocol described by Zhong et al. [79]. After 24 h of incubation in darkness, fluorescence was detected using a Zeiss LSM710 microscope (http://www.zeiss.com (accessed on 24 June 2024)), with the excitation and emission wavelengths set at 488 nm and 535 nm, respectively.

### 4.6. RNA Extraction and RNA-Seq Analysis

The RNA integrity was assessed using the RNA Nano 6000 Assay Kit on the Bioanalyzer 2100 system (Agilent Technologies, Santa Clara, CA, USA). Index-coded samples were clustered with the TruSeq PE Cluster Kit v3-cBot-HS (Illumina) on a cBot Cluster Generation System. After cluster generation, library preparations were sequenced on an Illumina Novaseq platform. The genome of *Petunia axillaris*, one of the parental species of *P. hybrida*, was used (https://solgenomics.net/ftp/genomes/Petunia_axillaris/assembly/Petunia_axillaris_v1.6.2_genome.fasta (accessed on 23 May 2024)). The paired-end clean reads were aligned to the reference genome with Hisat2 v2.0.5. FeatureCounts v1.5.0-p3 was used to count the number of reads mapped to each gene. Differential expression analysis between the two groups was conducted using the DESeq2 R package (1.20.0). Genes with an adjusted *p*-value < 0.05, as identified by DESeq2, were considered differentially expressed. A corrected *p*-value of 0.05 and an absolute fold change of 2 were set as the thresholds for significant differential expression. RNA-seq analysis was performed by Novogene Bioinformatics [80].

### 4.7. Measurements of Phytohormones

Fresh corollas (1.5 g) from *P. hybrida*, including *PhARF5*- and *PhARF19a*-silenced petunias and controls, were collected and extracted using a methanol mixture with an internal standard. Phytohormones were detected and analyzed with the ExionLC UPLC system (AB Sciex, Framingham, MA, USA) equipped with an Acquity UPLC^®^ CSH C18 column (1.7 µm, 2.1 × 150 mm, Waters), as previously described. The analysis was conducted using three biological replicates.

### 4.8. Statistical Analyses

Statistical analyses were conducted using a one-way analysis of variance (ANOVA), followed by Duncan’s multiple range test (DMRT) for comparisons involving more than two groups, and a Student’s *t*-test for pairwise comparisons, with at least three replicates. Furthermore, *p*-values ≤ 0.05 were considered statistically significant and marked with an asterisk on the comparison group.

## 5. Conclusions

This study highlights the crucial roles of *PhARFs*, particularly *PhARF5* and *PhARF19a*, in regulating flower development in *P. hybrida* through phytohormone metabolism and signaling pathways. We identified 29 PhARFs in *P. hybrida*, classified into four major clades, with flower development-related PhARFs dispersed across these clades. Silencing *PhARF5* and its close homolog *PhARF19a* resulted in significant alterations in corolla morphology, demonstrating their non-redundant roles. The transcriptomic analysis revealed that silencing these two homologous genes led to similar pathway changes, such as central carbon and hormone metabolism, plant hormone signal transduction, stress responses, and reproductive mechanisms. Both *PhARF5* and *PhARF19a* significantly impacted phytohormone biosynthesis, each triggering specific broad effects, emphasizing their involvement in complex feedback mechanisms. Furthermore, these two *PhARFs*, with their functional differences, are involved in crosstalk with other phytohormones, each gene affecting the hormone signaling pathways in distinct ways. Overall, our findings highlight the phylogenetic characteristics of the ARFs involved in flower development and provide insights into the commonalities and differences in the regulation of flower development by the non-redundant homolog pairs PhARF5 and PhARF19a.

## Data Availability

Reference genomes of *P. hybrida* were obtained from the public Genome website (https://solgenomics.net/ftp/genomes/Petunia_axillaris/assembly/Petunia_axillaris_v1.6.2_genome.fasta (accessed on 23 May 2024)). Transcriptomics data of *PhARF5-*, *PhARF19a*-silenced petunias, and controls have been uploaded to the NCBI BioProject database under accession number PRJNA1183582 (https://dataview.ncbi.nlm.nih.gov/object/PRJNA1183582 (accessed on 1 December 2023)) as required.

## Supplementary Materials

The following supporting information can be downloaded at: https://www.mdpi.com/article/10.3390/ijms252212249/s1.
