# Supplementary material for: Functional Divergence of the Closely Related Genes PhARF5 and PhARF19a in Petunia hybrida Flower Formation and Hormone Signaling
Source: Int J Mol Sci. 2024 Nov 14;25(22):12249. doi: 10.3390/ijms252212249 (PMC11594976; doi:10.3390/ijms252212249)
Supplement: Supplementary file 1 [file ijms-25-12249-s001.zip › Supplementary Figure.pdf]

Supplementary Figures

Title: Functional divergence of the homologous gene pair *PhARF5* and *PhARF19a* in *Petunia hybrida* flower formation and hormone signaling

Yiqing Ding[1#], Yunfeng Miao[1#], Lingxuan Huang[1#], Huijun Zhu[1], Wenle Li[1], Wei Zou[1], Shumin Yu[1], Bin Dong[1]✉, Shiwei Zhong[1]✉

Name of correspondence author: Bin Dong and Shiwei Zhong

Address: School of Landscape Architecture, Zhejiang Agriculture and Forestry University, Hangzhou 311300, Zhejiang, China

E-mail: dongbin@zafu.edu.cn, zsw1105@zafu.edu.cn

Supplementary information: 7 figures

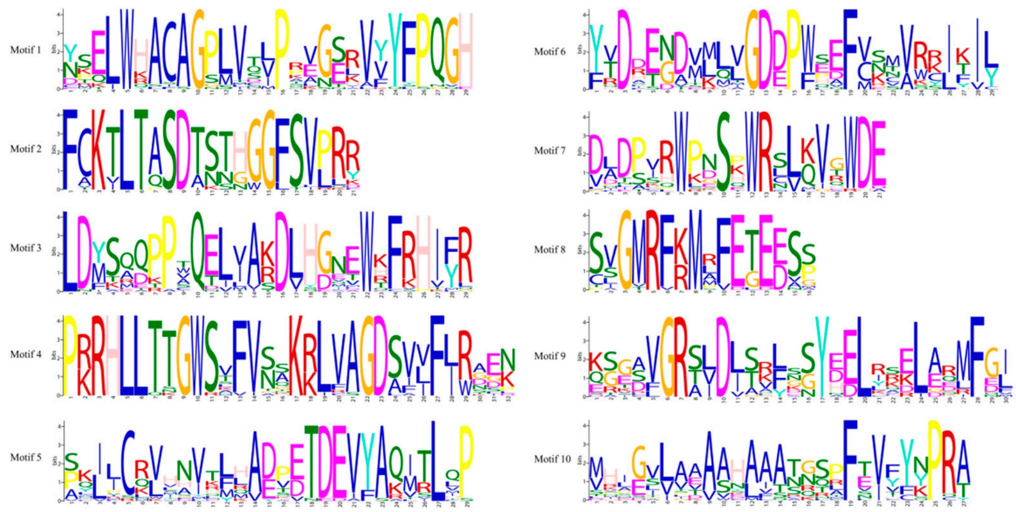

Figure S1. Conserved Motif of ARF proteins.

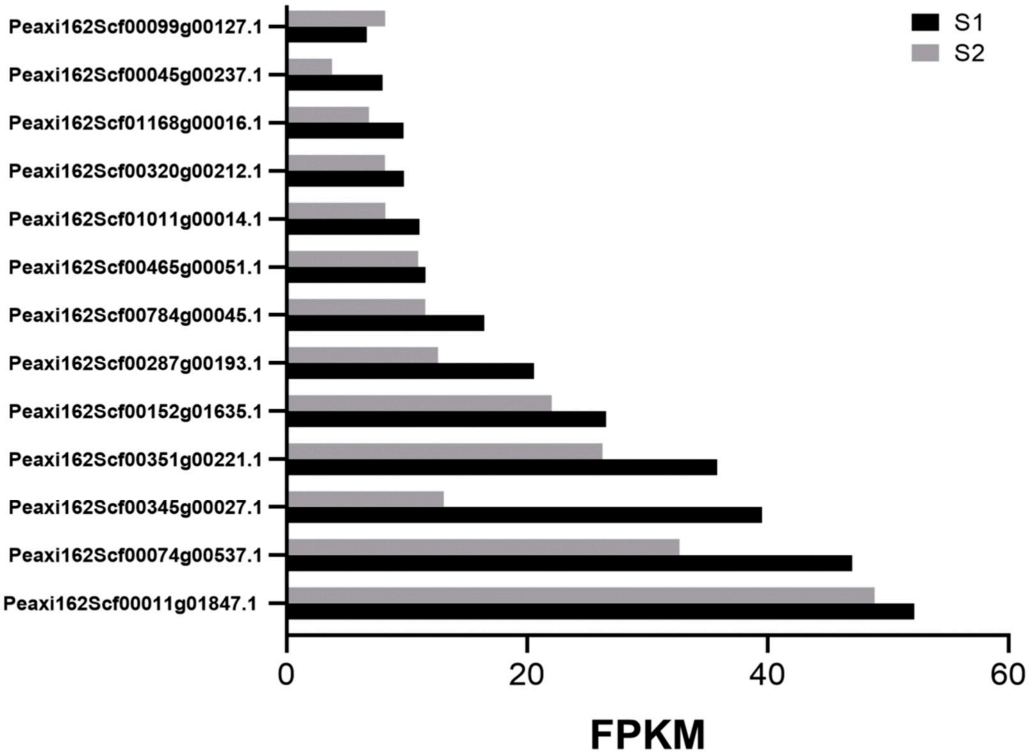

Figure S2. FPKM analysis of 12 *PhARFs* highly expressed in petunia corollas at two bud stages.

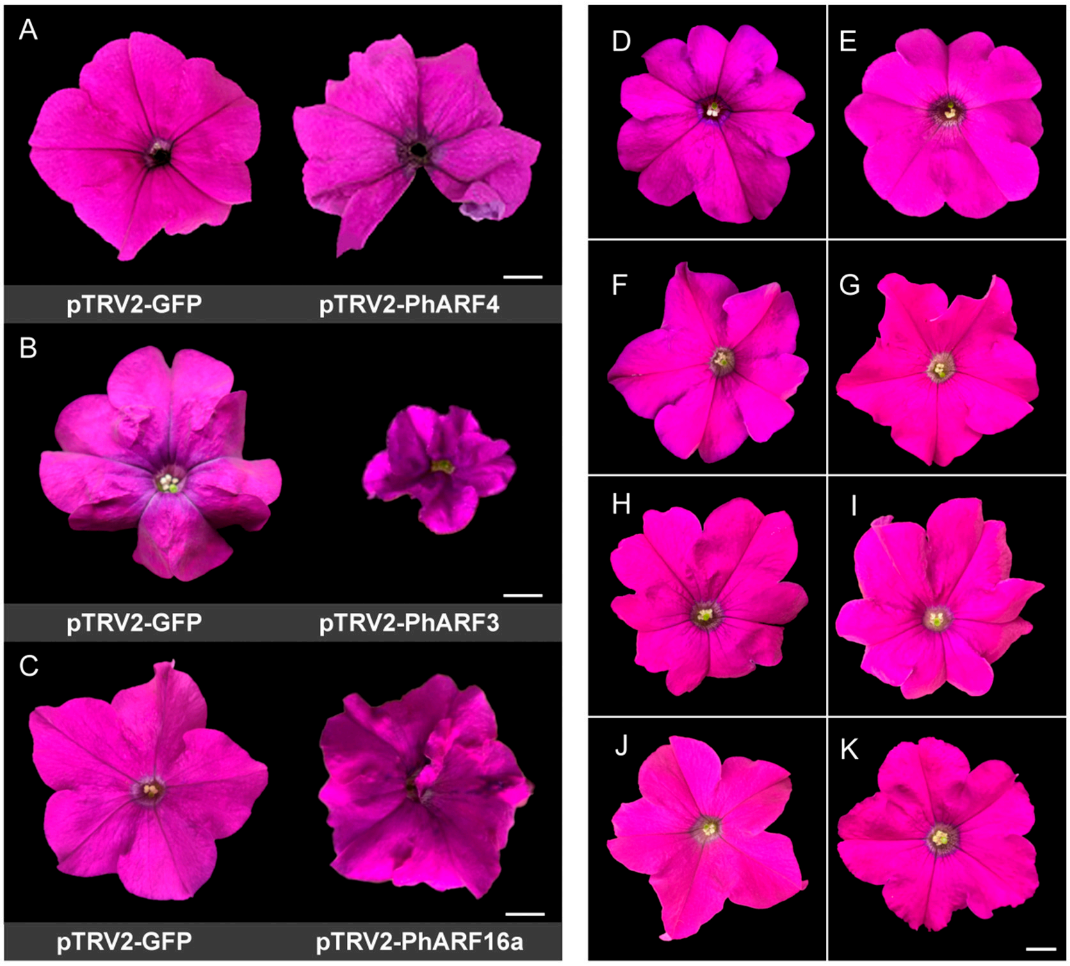

Figure S3. Figure S4. Effects of silencing *PhARF* genes on petunia corolla morphology. (A-C) Representative phenotypes of petunia corollas following VIGS-mediated silencing of *PhARF4* (*Peaxi162Scf00351g00221.1*) (A), *PhARF3* (*Peaxi162Scf00099g00127.1*) (B), and *PhARF16a* (*Peaxi162Scf01011g00014.1*) (C) compared to pTRV2-GFP control. Scale bars represent 1 cm; (D-K) Representative phenotypes of petunia corollas following controls (D), and VIGS-mediated silencing of *PhARF1a* (*Peaxi162Scf00011g01847.1*) (E), *PhARF2a* (*Peaxi162Scf01168g00016.1*) (F), *PhARF6a* (*Peaxi162Scf00465g00051.1*) (G), *PhARF6b* (*Peaxi162Scf00045g00237.1*) (H), *PhARF8a* (*Peaxi162Scf00784g00045.1*) (I), *PhARF11* (*Peaxi162Scf00074g00537.1*) (J), *PhARF18* (*Peaxi162Scf00345g00027.1*) (K), Scale bars represent 1 cm.

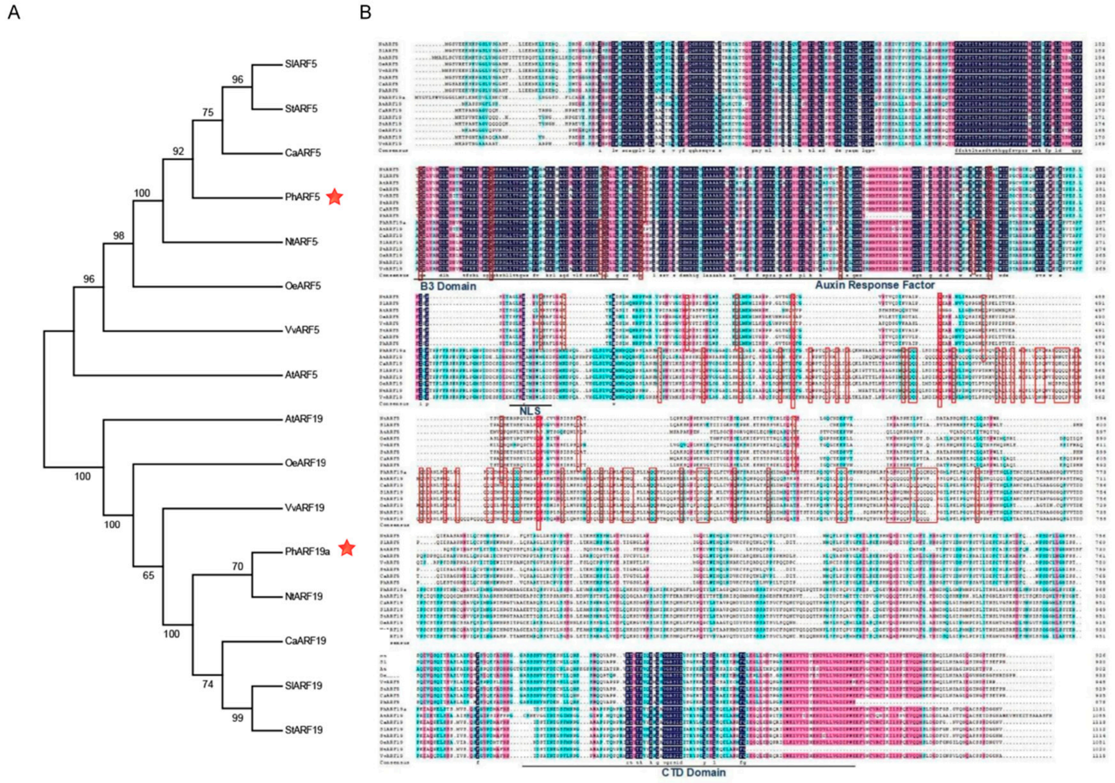

Figure S4. Phylogenetic analysis and protein sequence alignment of PhARF5 and PhARF19a. (A) Phylogenetic analysis of PhARF5, PhARF19a, and ARF5 and ARF19a reported in other species, including *S. lycopersicum* SlARF5 (NP_001234545.1) and SlARF19a (NP_001234740.2), *Nicotiana tabacum* NtARF5 (XP_016465083.1) and NtARF19a (XP_016515513.1), *Arabidopsis thaliana* AtARF5 (NP_001321214.1) and AtARF19a (NP_173356.1), *Vitis vinifera* VvARF5 (XP_003634382.2) and VvARF19a (XP_010656700.1), *Solanum tuberosum* StARF5 (XP_006342026.1) and StARF19a (XP_006365636.1), *Capsicum annuum* CaARF5 (XP_047267293.1) and CaARF19a (XP_016580257.2), and *Olea europaea* OeARF5 (XP_022859505.1) and OeARF19a (XP_022883057.1); (B) Alignment of the deduced amino acid sequence of PhARF5, PhARF19a, and ARF5 and ARF19a reported in other species. Amino acid residues identical to that of PhARF5 and PhARF19a are shadowed with colors. The glutamate (Q)-rich middle region is marked by red boxes.

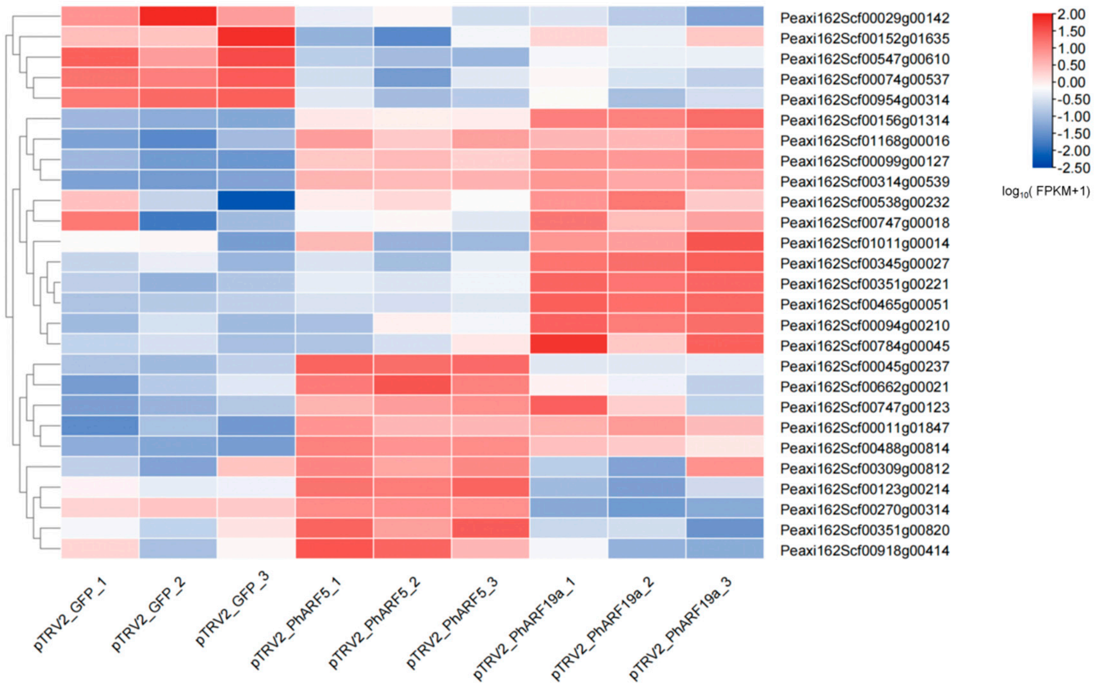

Figure S5. Heatmaps of *PhARFs* expression profiles in *PhARF5*- and *PhARF19a*-silenced petunias and controls.

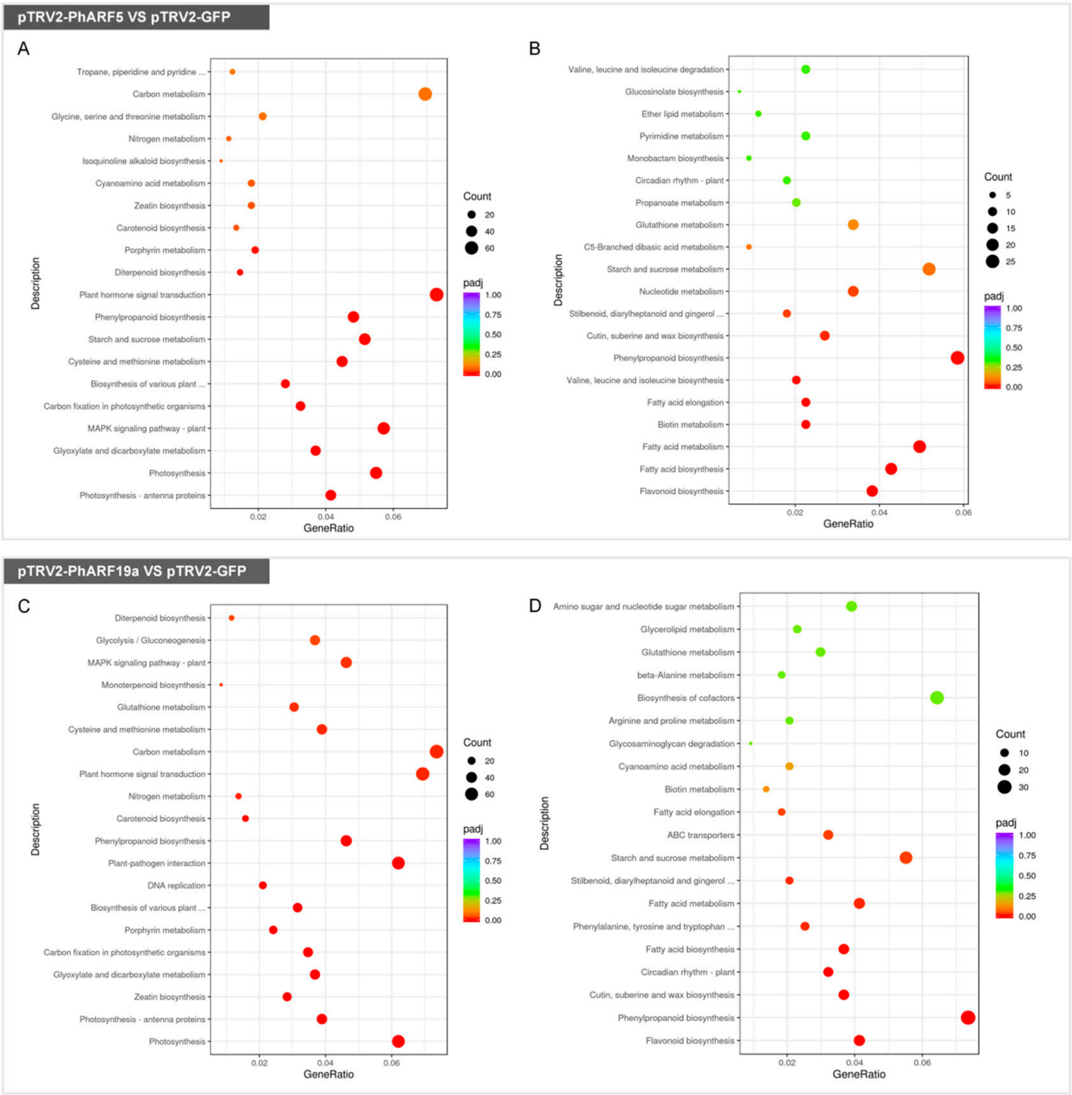

Figure S6. Analysis of the differential expressed genes in *PhARF5*- and *PhARF19a*-silenced petunia corollas compared with controls. (A-B) KEGG analysis indicated that the enrichment of members was significantly upregulated (A) and significantly downregulated (B) between *PhARF5*-silenced petunia corollas compared with controls (upper). (C-D) Members were significantly upregulated (C) and significantly downregulated (D) between *PhARF19a*-silenced petunia corollas compared with controls.

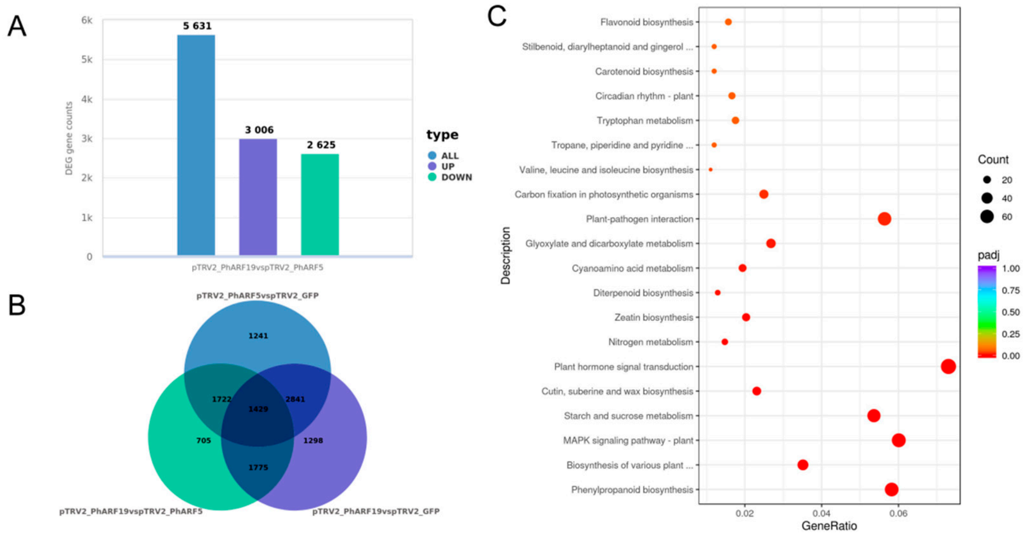

Figure S7. Transcriptome analysis of *PhARF5*- compared with *PhARF19a*-silenced petunias. (A) Bar chart of differentially expressed genes (DEGs) in *PhARF5*-silenced compared with *PhARF19a*-silenced petunias; (B) Venn diagram showing the overlap of DEGs pairwise comparisons among *PhARF5*- and *PhARF19a*-silenced petunias and controls; (C) KEGG analysis indicated that the enrichment of DEGs between *PhARF5*-silenced and *PhARF19a*-silenced petunias.
